# Identification of distinct symptom profiles in primary brain tumor patients: a prospective longitudinal study

**DOI:** 10.1186/s12883-025-04595-6

**Published:** 2025-12-26

**Authors:** Rongqing Li, Zikai Zhang, Xin Zhang, Jiefang Song, Yawen Wu, Linzhi Wu, Sailu Mao, Jinxia Jiang, Li Zeng

**Affiliations:** 1 Department of Neurosurgery, Tongji Hospital, School of Medicine, Tongji University, Shanghai, People’s Republic Of China; 2 Department of Science Administration, Tongji Hospital, School of Medicine, Tongji University, Shanghai, People’s Republic Of China; 3 Department of Neurosurgery, Shanghai Tenth People’s Hospital, School of Medicine, Tongji University, Shanghai, People’s Republic Of China; 4 Department of Nursing, Tongji Hospital, School of Medicine, Tongji University, Shanghai, People’s Republic Of China

**Keywords:** Symptom profile, Primary brain tumor, Latent profile analysis

## Abstract

**Background:**

Symptom burden in primary brain tumor patients varies, emphasizing the need for comprehensive understanding to improve patient care. This study aims to identify distinct symptom clusters among brain tumor patients in Shanghai, China, using Latent Profile Analysis (LPA) to guide personalized diagnosis, treatment, and supportive care.

**Methods:**

A longitudinal study was conducted among 161 patients with primary brain tumors in Shanghai. Participants completed the MD Anderson Symptom Inventory Brain Tumor Module (MDASI-BT) at three intervals: the day of admission (T1), three days after surgery (T2), and two weeks after surgery (T3). Latent Profile Analysis (LPA) was used to identify subgroups with unique symptom patterns.

**Results:**

Six distinct subgroups were identified (entropy = 0.964), ranging from low-burden to persistently severe patterns. Subgroup membership was partially associated with age, tumor grade, and diagnosis. These subgroups were: transient postoperative burden group, stable symptom with cognitive emergence group, distress-predominant, low burden group, elderly–high grade, persistently severe group, nausea-dominant recovery group, and distress-plus-nausea, younger urban group.

**Conclusion:**

Our findings reveal substantial heterogeneity in perioperative symptom experiences among brain tumor patients. Identifying subgroups with high and persistent symptom burden may help clinicians target interventions such as enhanced education, proactive monitoring, rehabilitation, psychological support, and antiemetic management. This subgroup-based approach may improve quality of life, reduce morbidity, and guide precision supportive care in neuro-oncology.

**Supplementary Information:**

The online version contains supplementary material available at 10.1186/s12883-025-04595-6.

## Background

Brain tumors, as complex and heterogeneous neoplasms, can present with various symptoms, causing significant morbidity and mortality worldwide. Despite the relatively low overall incidence rate of brain tumors (10.82 per 100, 000 person-years worldwide), the five-year survival rate remains only 36% [1, 2]. They manifest with a broad spectrum of symptoms, most commonly headache, seizures, cognitive impairment, motor weakness, visual disturbances, language dysfunction, and emotional distress [3, 4]. The prevalence and severity of these symptoms vary considerably according to tumor location, histopathology, World Health Organization (WHO) grade, and the degree of mass effect.

Brain tumor treatment options encompass surgery, radiotherapy, chemotherapy, immunotherapy, traditional Chinese medicine, or combination therapies. Among them, surgery stands out as the primary choice, performing well in removing diseased tissue, alleviating compression symptoms, and preserving neurological function [5]. However, the diagnosis and management of brain tumors present substantial clinical challenges due to the diverse symptomatology and variable disease courses observed among affected individuals [6–8]. These symptoms often occur in clusters rather than isolation, reflecting shared underlying mechanisms such as tumor-related mass effect, neuroinflammation, and treatment-related toxicity. Identifying and understanding such symptom clusters is clinically important, as it can facilitate early detection of high-risk patients, enable targeted symptom management, and potentially improve quality of life, functional outcomes, and survival.

While previous research has yielded valuable insights into the clinical manifestations of brain tumors, the inherent diversity in symptom profiles among patients still implies the presence of distinct subgroups or latent classes within the population [3, 9, 10]. Furthermore, there is a lack of longitudinal studies in Chinese brain tumor patients that track dynamic symptom changes at multiple perioperative time points.

Latent Profile Analysis (LPA) offers a statistical framework for identifying subgroups based on shared symptom patterns, without prior assumptions about the number of subgroups or their composition [11, 12]. Although LPA has been successfully applied to characterize symptom heterogeneity in other cancer populations [13], it has rarely been used to investigate longitudinal changes in symptom clusters among brain tumor patients.

Therefore, the present prospective longitudinal study aimed to identify distinct symptom profiles in primary brain tumor patients at three critical perioperative time points—on admission, three days after surgery, and two weeks after surgery—using LPA. By identifying subgroups of patients with distinct symptom patterns, LPA enables the recognition of population-level heterogeneity, providing a foundation for subgroup-specific monitoring and supportive strategies.

## Methods

### Study design

This prospective longitudinal study was designed to include all patients with primary brain tumors who sought surgical treatment at the Department of Neurosurgery, Shanghai Tongji Hospital, between September 2023 and March 2024. Ethical approval for the study was obtained from the Ethical Committee of Shanghai Tongji Hospital prior to its commencement. Symptom data were collected at three perioperative time points: the day of admission (T1), three days after surgery (T2), and two weeks after surgery (T3).

### Participants

**Fig. 1:**
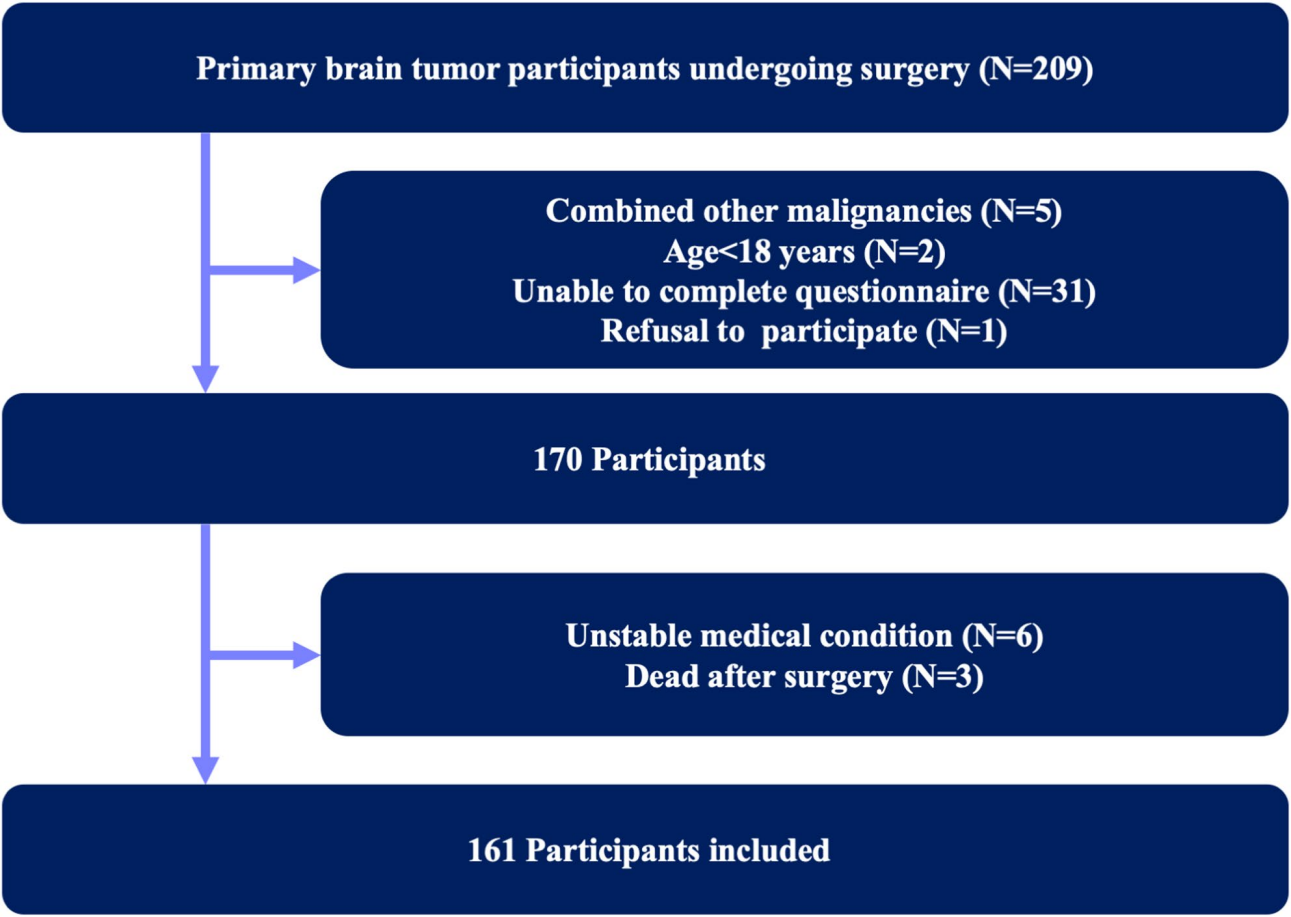
Participants recruitment protocol

The inclusion criteria for the study were: (i) patients with a primary brain tumor meeting the diagnostic criteria of the 2016 WHO Classification of Tumors of the Central Nervous System [14] (which was the standard in our institution during the study period; the 2021 WHO classification was not yet routinely adopted); (ii) those undergoing brain tumor surgery; (iii) those aged ≥ 18 years; (iv) those capable of communicating normally at all three time points; and (v) those who volunteered to participate in this study. The exclusion criteria for the study included: (i) patients with a clinical diagnosis of combined other malignancies, psychiatric disorders, or severe cognitive impairment; (ii) those unable to complete questionnaire; or (iii) unstable medical conditions, defined as severe systemic illness (e.g., uncontrolled infection, decompensated cardiopulmonary disease) or perioperative complications preventing participation, as well as patients who died before completing the follow-up (Fig. 1).

Regarding patients with aphasia, those with mild language dysfunction who could understand and respond (with assistance if necessary) were included; patients with severe aphasia unable to complete the questionnaire were excluded.

### Measurements

Participants’ general characteristics, such as gender, age, marital status, years of education, and income, were collected through a self-reported questionnaire. Disease-related data were extracted from their medical records.

MD Anderson Symptom Inventory Brain Tumor Module (MDASI-BT)was used to evaluate the severity of the patients’ symptoms at three time points (T1, T2, and T3). The scale, developed by researchers at the MD Anderson Cancer Center, serves as a valuable tool for clinicians and researchers to comprehend the unique challenges and symptoms encountered by patients suffering from brain tumors [15]. The MDASI-BT consists of 13 core symptom items (e.g., pain, fatigue, nausea, disturbed sleep), 9 brain tumor-specific symptom items (e.g., seizures, visual disturbance, weakness, speech difficulty), and 6 symptom distress items. Each item is rated on a 0–10 numeric scale, where higher scores indicate greater symptom severity or interference. The complete list of MDASI-BT items used in this study is provided in Supplementary Table S1.

Of the patients initially enrolled, only those who completed the MDASI-BT at all three time points were included in the final analysis. Patients with incomplete data at any time point were excluded (complete-case analysis).

### Statistical analysis

Descriptive statistics were used to summarize demographic and clinical variables. Continuous variables were reported as means ± standard deviations if normally distributed, or medians with interquartile ranges otherwise. Categorical variables were expressed as frequencies and percentages. Group differences were examined using ANOVA or Kruskal–Wallis tests for continuous variables and χ² tests for categorical variables.

Latent Profile Analysis, also known as finite mixture modeling, was applied to classify patients into subgroups according to their MDASI-BT symptom profiles across the three time points (T1, T2, T3). LPA identifies homogeneous subgroups based on continuous indicators without imposing arbitrary cut-off scores. Models with increasing numbers of classes were compared using log-likelihood, Akaike Information Criterion (AIC), Bayesian Information Criterion (BIC), entropy, and the Lo–Mendell–Rubin (LMR) test [16]. The optimal solution was determined by the balance of statistical fit indices, interpretability, and parsimony.

After the model was selected, classes were named according to their most salient characteristics and temporal patterns of symptom change. This naming process was based on both quantitative results (symptom scores, trajectory patterns) and clinical interpretability. All analyses were conducted using Stata version 15.0 SE (StataCorp LLC, TX, USA).

## Results

### Participants characteristics

A total of 161 patients with primary brain tumors completed all three assessments and were included in the analysis. The mean age was 52.0 years (SD = 13.2), and 56.8% were male. The majority were married (88.3%), had completed high school or college education (85.2%), and resided in urban areas (75.9%). Regarding income, 42.6% reported a monthly household income between $3000 and $5000, while 23.5% reported more than $5000.

With respect to tumor characteristics, meningiomas (41.4%) and gliomas (30.9%) were the most common histological types, followed by pituitary tumors (8.0%) and nerve sheath tumors (8.6%). More than half of the tumors were stage I (59.9%). Supratentorial tumors accounted for 63.6%, most frequently in the frontal (16.7%) and parietal (9.3%) lobes. Infratentorial tumors comprised 14.8%, including 8.6% located in the brainstem and 6.2% in the cerebellum. Multiple or midline/central tumors were observed in 21.0% of cases. Detailed demographic and clinical characteristics are summarized in Table 1.

**Table 1:** Demographics and clinical characteristics of the participants (*N* = 161)

Demographics and clinical characteristic		Frequency(%)
Gender		
	Male	92(56.79%)
	Female	69(42.59%)
Age		52.01 (13.21)
Marriage status		
	Married	143(88.27%)
	Single/Divorced	18(11.11%)
Education		
	High school/associate/college	138(85.19%)
	Bachelor’s degree or above	24(14.81%)
Residence		
	Urban	123(75.93%)
	Rural area	35(21.6%)
Income		
	$1000~$3000	38(23.46%)
	$3000~$5000	69(42.59%)
	>$5000	38(23.46%)
Cancer stage		
	I	97(59.88%)
	II	35(21.6%)
	III	29(17.9%)
Cancer type		
	Glioma	50(30.86%)
	Meningioma	67(41.36%)
	Pituitary tumor	13(8.02%)
	Nerve sheath tumor	14(8.64%)
	Pineal tumor	3(1.85%)
	Others	14(8.64%)
Tumor location		
	Supratentorial	103(63.58%)
	Insula	2(1.23%)
	Parietal	15(9.26%)
	Frontal	27(16.67%)
	Temporal	11(6.79%)
	Deep nuclei	6(3.7%)
	Occipital	8(4.94%)
	Midline/Central	34(20.99%)
	Infratentorial	24(14.81%)
	Brainstem	14(8.64%)
	Posterior fossa—Cerebellum	10(6.17%)
	Multiple	34(20.99%)

## LPA results

Latent profile analyses were conducted with two, three, four, five, six and seven subgroups, with model fit statistics shown in Table 2. Considering the low log-likelihood, AIC, and BIC values, as well as a relatively high entropy (0.964) and a LMR test with P-value < 0.001 of the model, a model comprising six subgroups was finally selected.

**Table 2:** Model fit statistics

Fit models	1 class	2 class	3 class	4 class	5 class	6 class	7 class
Log-likelihood	-1978.125	-1926.481	-1905.536	-1892.808	-1902.546	-1874.984	-1872.167
Degrees of freedom	6	10	14	18	22	26	30
AIC	3968.251	3872.962	3839.071	3821.615	3849.093	3801.967	3804.335
BIC	3986.665	3903.651	3882.036	3876.856	3916.609	3881.759	3896.402
Entropy	NA	0.932	0.939	0.802	0.807	0.964	0.964
LMR test	NA	96.917	39.306	23.885	-18.276	51.724	5.285
LMR test, P-value	NA	< 0.001	< 0.001	0.0012	1	< 0.001	0.63

## MDASI-BT score at three time-points among subgroups

**Fig. 2:**
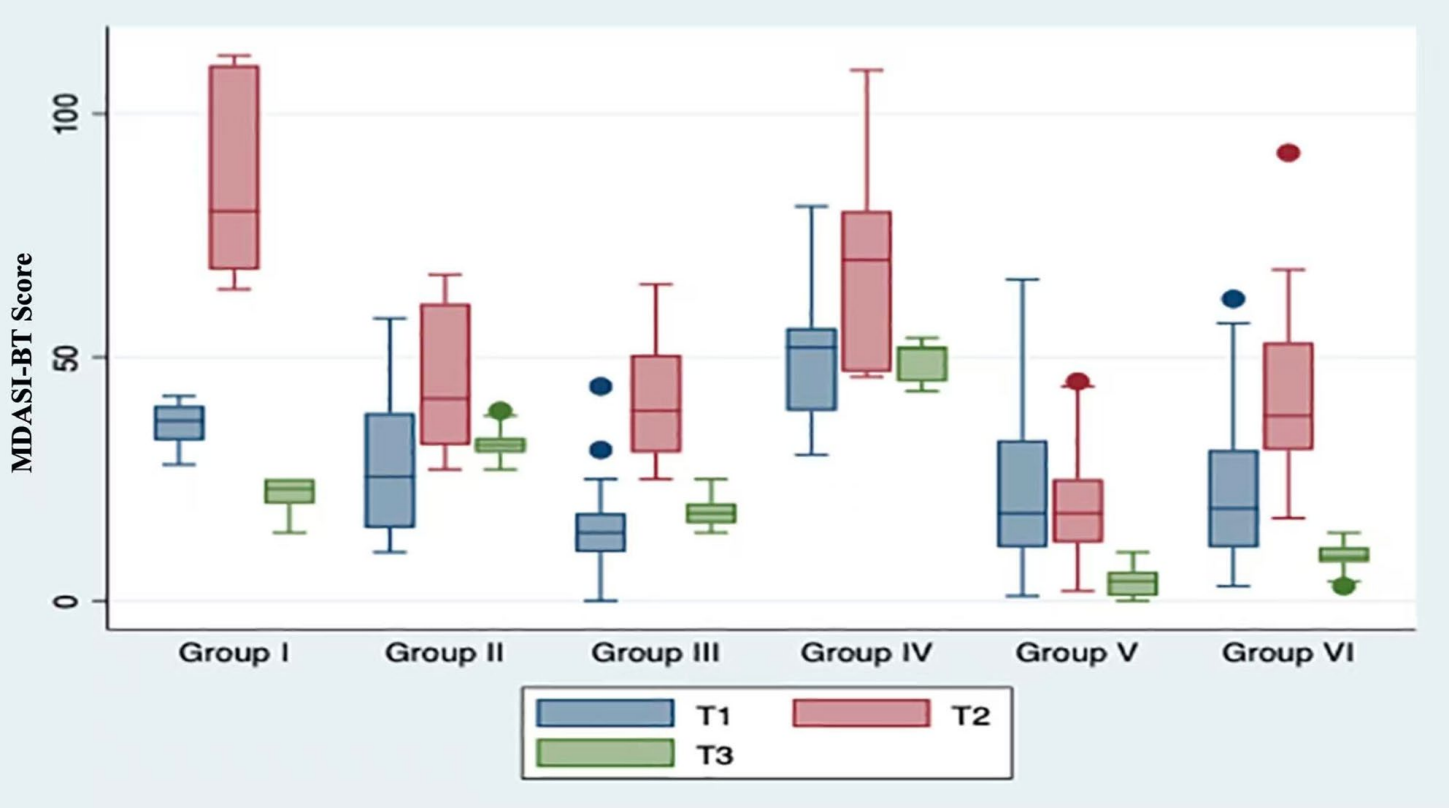
MDASI-BT score distribution among different subgroups

Group I exhibited an initial MDASI-BT score of approximately 36 at T1. This score notably increased at T2 but then declined to around 20 at T3, which was lower than the score recorded at T1. Group II displayed minimal score variance across the three time points, hovering around 30. In contrast, Group III consistently scored below 20 at T1 and T3, with marginal elevation at T2. Group IV exhibited a substantial burden, consistently scoring above 50 throughout. Group V consistently scored around 20 at both T1 and T2, but experienced a significant drop to 3.66 at T3. Lastly, Group VI began at approximately 20 at T1, and surged to 41 at T2, before declining below 10 at T3 (Fig. 2, Table 3).

**Table 3:** MDASI-BT score in different subgroups

	T1	T2	T3	*P*-value
Group I (*N* = 7)	36 (4.62)	83.86 (19.95)	21.57 (3.95)	0.035
Group II (*N* = 16)	27.82 (14.03)	45.94 (14.74)	32.13 (3.34)	0.383
Group III (*N* = 20)	15.2 (10.08)	41.7 (12.59)	18.4 (2.85)	0.187
Group IV (*N* = 5)	51.6 (19.42)	70.4 (26.10)	49.2 (4.86)	0.831
Group V (*N* = 61)	21.54 (13.81)	19.18 (10.02)	3.66 (2.54)	< 0.001
Group VI (*N* = 52)	22.26 (14.15)	41.34 (15.22)	9.3 (2.74)	< 0.001

P-values indicate differences in MDASI-BT scores across the three time points (T1, T2, and T3) within each subgroup

### Demographic and clinical characteristics of subgroups

Table 4 displays the demographic and clinical characteristics across six subgroups.

**Table 4:** Demographic and clinical characteristics in different subgroups

	Group I (*N* = 7)		Group II (*N* = 16)	Group III (*N* = 20)	Group IV (*N* = 5)	Group V (*N* = 61)		Group VI (*N* = 52)
Gender								
Male	4 (57.14%)		8 (50%)	13 (65%)	3 (60%)	33 (54.10%)		31 (59.62%)
Female	3 (42.86%)		8 (50%)	7 (35%)	2 (40%)	28 (45.90%)		21 (40.38%)
Age	50.72 (11.70)	50.13 (15.61)		48.2 (15.21)	62 (11.94)	54.11 (11.70)		50.79 (13.32)
Marriage Status								
Married	7 (100%)		15 (93.75%)	16 (80%)	5 (100%)	56 (91.80%)		44 (84.62%)
Single/ Divorced	0		1 (6.25%)	4 (20%)	0	5 (8.19%)		8 (15.38%)
Education								
High school/associate/college	6 (85.71%)		13 (81.25%)	17 (85%)	5 (100%)	53 (86.88%)		44 (84.62%)
Bachelor’s degree or above	1 (14.29%)		3 (18.75%)	3 (15%)	0	8 (13.11%)		8 (15.38%)
Residence								
Urban	4 (57.14%)		11 (68.75%)	15 (75%)	1 (20%)	50 (81.97%)		42 (80.77%)
Rural area	3 (42.86%)		5 (31.25%)	2 (25%)	4 (80%)	11 (18.03%)		10 (19.23%)
Income								
$1000~$3000	3 (42.86%)		4 (25%)	5 (25%)	4 (80%)	13 (21.31%)		9 (17.31%)
$3000~$5000	4 (57.14%)		7 (43.75%)	9 (45%)	1 (20%)	28 (45.90%)		20 (38.46%)
>$5000	0		3 (18.75%)	4 (20%)	0	16 (26.23%)		15 (28.85%)
Cancer stage								
I	4 (57.14%)		8 (50%)	12 (60%)	3 (60%)	36 (59.02%)		34 (65.38%)
II	0		6 (37.50%)	5 (25%)	0	14 (22.95%)		10 (19.23%)
III	3 (42.86%)		2 (12.50%)	3 (15%)	2 (40%)	11 (18.03%)		8 (15.38%)
Cancer type								
Glioma	4(57.14%)		6(37.5%)	9(45%)	2(40%)	14(22.95%)		15(28.85%)
Meningioma	3(42.86%)		8(50%)	8(40%)	2(40%)	22(36.07%)		24(46.15%)
Pituitary tumor	0		0	0	1(20%)	9(14.75%)		3(5.77%)
Nerve sheath tumor	0		1(6.25%)	2(10%)	0	5(8.2%)		6(11.54%)
Pineal tumor	0		1(6.25%)	1(5%)	0	1(1.64%)		0
Others	0		0	0	0	10(16.39%)		4(7.69%)
Tumor location								
Supratentorial	7(100%)	12(75%)		12(60%)	5(100%)	39(64%)	28(53.85%)	
Insula	0	1(6.25%)		0	0	1(2%)	0	
Parietal	3(42.86%)	1(6.25%)		3(15%)	0	5(8.2%)	3(5.77%)	
Frontal	1(14.29%)	3(18.75%)		1(5%)	3(60%)	12(19.67%)	7(13.46%)	
Temporal	0	2(12.5%)		1(5%)	0	4(6.56%)	4(7.69%)	
Deep nuclei	2(28.57%)	2(12.5%)		0	0	1(1.64%)	1(1.92%)	
Occipital	1(14.29%)	0		1(5%)	0	3(4.92%)	3(5.77%)	
Midline/Central	0	3(18.75%)		6(30%)	2(40%)	13(21.31%)	10(19.23%)	
Infratentorial	0	2(12.5%)		2(10%)	0	10(16.39%)	10(19.23%)	
Brainstem	0	1(6.25%)		2(10%)	0	6(9.84%)	5(9.62%)	
Posterior fossa—Cerebellum	0	1(6.25%)		0	0	4(6.56%)	5(9.62%)	
Multiple	0	2(12.5%)		6(30%)	0	12(19.67%)	14(26.92%)	

The six latent groups differed significantly in demographic, socioeconomic, and clinical characteristics (Table 4). Group IV had the oldest mean age (62 years) and the highest proportion of rural residence (80%) and low household income ($1000–3000, 80%). In contrast, Group VI included younger patients, predominantly urban dwellers, and those with higher incomes.

Tumor-related factors also varied across groups. Gliomas were more frequent in Group I and III, while meningiomas predominated in Group VI. High-grade (stage III) tumors were relatively more common in Group I and IV, both showing higher symptom burdens. Tumor location also showed subgroup trends: patients with midline/central and multiple lesions were more concentrated in Group III and V, whereas infratentorial and brainstem tumors were slightly more frequent in Group V and VI. These findings suggest that both sociodemographic and tumor-related characteristics contribute to the heterogeneity of perioperative symptom experiences.

### Symptom occurrence and severity of subgroups

**Fig. 3:**
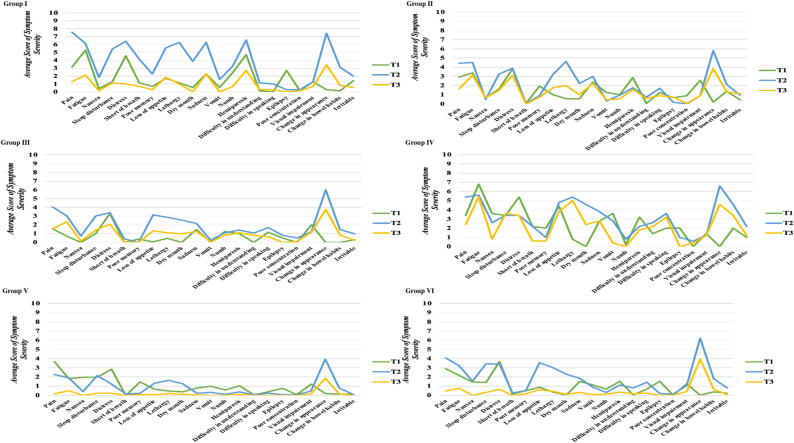
Symptom occurrence and severity in different subgroups

Distinct temporal patterns of symptom severity were observed across the six latent subgroups, as summarized in Table 5. For example, some groups (e.g., Group I, Group II) showed peaks at specific perioperative time points, whereas others (e.g., Group IV) exhibited persistently high symptom burden. Detailed subgroup symptom characteristics are presented in Table 5, Fig. 3.

**Table 5:** Symptom occurrence and severity in different subgroups

Group	Key Characteristics
Group I	• Notable increase in sickness-related symptoms at T2 (pain, distress, dyspnea, sadness, dry mouth, poor appetite, hemiparesis, appearance changes). • Notable increase in fatigue-related symptoms at T2 (fatigue, sleep disturbance, lethargy, numbness). • Gradual relief by T3.
Group II	• Prominent fatigue & sickness-related symptoms at T1 (distress, hemiparesis, sadness, vision problems). • Reduction in some original symptoms (e.g., hemiparesis) but emergence of new symptoms (e.g., poor memory, lethargy) by T2/T3. • Overall burden relatively stable.
Group III	• Strong distress at T1, persisted at T2. • Increase Overall burden at T2, decrease at T3. • Lowest overall symptom burden among subgroups.
Group IV	• Consistently high severity across all symptoms and time points. • Highest global burden.
Group V	• Nausea prominent at T1. • Burden stable T1–T2. • Overall severity became the lowest among all subgroups; almost all symptoms disappeared by T3.
Group VI	• Similar symptom pattern to Group III, with distress being most prominent at T1. • Distinctive nausea symptoms. • Nausea symptoms were alleviated by T3.

### Naming of subgroups

To enhance clinical interpretability, each latent subgroup was labeled according to its predominant symptom patterns in conjunction with demographic and clinical characteristics. Group I, characterized by transient postoperative increases in sickness- and fatigue-related symptoms with subsequent recovery, was named the transient postoperative burden group. Group II demonstrated relatively stable overall burden but the emergence of cognitive-related symptoms (e.g., poor memory, lethargy) after surgery, leading to the label stable symptom with cognitive emergence group. Group III was dominated by emotional distress with otherwise low overall symptom burden and a higher proportion of urban, better-educated patients, thus termed the distress-predominant, low burden group. Group IV exhibited persistently high severity across almost all symptoms and included older patients with more advanced tumors, leading to the designation elderly–high grade, persistently severe group. Group V was characterized by prominent nausea at baseline but substantial recovery by T3. Notably, this subgroup included the highest proportion of patients with low-grade and benign tumors (e.g., meningioma, pituitary), consistent with their favorable recovery trajectory. It was therefore named the nausea-dominant recovery (predominantly low-grade, favorable prognosis) group. Finally, Group VI showed a symptom profile similar to Group III but with additional nausea symptoms, often among younger, urban patients, and was therefore designated the distress-plus-nausea, younger urban group.

## Discussion

This prospective study identified six distinct symptom subgroups among patients with primary brain tumors using latent profile analysis across three perioperative time points. The findings highlight substantial heterogeneity in symptom occurrence and severity, suggesting that symptom experiences are not uniform but follow specific trajectories. To our knowledge, this is one of the first longitudinal studies to apply latent profile analysis in this population, offering a more nuanced understanding of how symptom clusters evolve after craniotomy.

Compared with prior studies in other cancer populations, such as breast and lung cancer, where latent groups often captured fatigue-dominant or psychological distress–dominant subgroups [17–20], our study revealed patterns unique to brain tumor patients. For instance, the nausea-dominant recovery subgroup and the cognitive-emergence subgroup reflect the particular neurocognitive and perioperative complications associated with brain tumors and neurosurgical treatment. These results underscore the importance of studying this population separately rather than extrapolating from other malignancies.

Several demographic and clinical characteristics appeared to align with subgroup membership. The persistently severe subgroup (Group IV) included older patients and those with more advanced disease, while the nausea-dominant recovery subgroup (Group V) was composed primarily of patients with low-grade or benign tumors such as meningioma and pituitary adenoma. This suggests that tumor histopathology, grade, and location may partially explain differences in symptom trajectories. Indeed, prior research has shown that symptoms such as seizures, cognitive dysfunction, or aphasia often vary depending on tumor region and mass effect [21–23]. While our study did not have sufficient sample size to conduct systematic stratified analyses by diagnosis or tumor site, these factors likely represent important sources of heterogeneity and warrant investigation in future multi-center studies.

The clinical implications of our findings are notable. First, identifying high-burden groups, such as the elderly–high grade, persistently severe subgroup, highlights the need for comprehensive supportive strategies. For these patients, interventions may include enhanced patient education, proactive symptom monitoring, and multidisciplinary supportive care to reduce symptom burden and prevent further deterioration [24, 25]. Second, recognizing subgroups at risk for emerging cognitive or psychological symptoms may facilitate earlier screening and rehabilitation. Finally, patients in favorable subgroups, such as the nausea-dominant recovery group, may benefit from reassurance and routine follow-up rather than intensive intervention, thereby optimizing resource allocation. These subgroup-based insights may guide more personalized, precision-oriented supportive care for brain tumor patients.

Potential interventions tailored to symptom subgroups include psychological support for distress-predominant groups, rehabilitation programs for patients with motor or cognitive impairments, enhanced education and proactive monitoring for the persistently severe group, and antiemetic or nutritional management for nausea-dominant groups [26, 27]. Incorporating such targeted strategies into clinical practice may improve quality of life, reduce morbidity, and support long-term survivorship in this population. Rather than focusing solely on individual-level personalization, these findings highlight the importance of subgroup-based interventions, where different patient classes may benefit from differentiated monitoring, rehabilitation, and supportive care approaches.

This study has several limitations. First, the sample size was modest, and subgroup distributions limited our ability to fully examine the effects of tumor grade, histopathology, and anatomical location. Second, we excluded patients unable to complete questionnaires at all time points, which may underestimate symptom burden in those with severe communication deficits such as aphasia. Third, patients with brain tumors who did not undergo surgery were not included; these individuals may differ meaningfully in symptom patterns and disease trajectory, and excluding them may limit the generalizability of our findings. Although systematic data on the proportion of conservatively treated patients are lacking, prior reports suggest wide variation by tumor type (e.g., ~ 40% for gliomas, ~ 8% for meningiomas) and sociodemographic factors [28, 29]. Fourth, the follow-up was limited to two weeks after surgery, and longer-term trajectories remain unknown. Future studies with larger multi-center cohorts, integration of tumor biology and neuroimaging data, and extended follow-up will be needed to validate and extend our findings.

## Conclusion

In conclusion, this study demonstrated six distinct and clinically meaningful symptom trajectories among brain tumor patients after craniotomy. By integrating symptom patterns with demographic and clinical features, we identified subgroups that may benefit from tailored screening, intervention, and follow-up strategies. These findings provide an initial framework for developing precision supportive care approaches in neuro-oncology.

## Supplementary Information

Supplementary Material 1.

## Data Availability

The datasets used and/or analyzed during the current study are available from the corresponding author on reasonable request.

## Abbreviations

MDASI-BT: MD Anderson Symptom Inventory Brain Tumor Module
LPA: Latent Profile Analysis
WHO: World Health Organization
AIC: Akaike Information Criterion
BIC: Bayesian Information Criterion
LMR: Lo–Mendell–Rubin
