# Supplementary material for: Identification of distinct symptom profiles in primary brain tumor patients: a prospective longitudinal study
Source: BMC Neurol. 2025 Dec 26;26:53. doi: 10.1186/s12883-025-04595-6 (PMC12849636; doi:10.1186/s12883-025-04595-6)
Supplement: Supplementary file 1 — Supplementary Material 1. [file 12883_2025_4595_MOESM1_ESM.docx]

Supplementary Table 1 MD Anderson Symptom Inventory Brain Tumor Module (MDASI-BT)

Part I How serve are your symptoms?

People with cancer frequently have symptoms that are caused by their disease or by their treatment. We asked you to rate how severe the following symptoms have been in the last 24hrs. Please select a number from 0 (symptom has not been present) to 10 (the symptom was as bad as you can imagine it could be) for each item.

| Item | 0 | 1 | 2 | 3 | 4 | 5 | 6 | 7 | 8 | 9 | 10 |
| --- | --- | --- | --- | --- | --- | --- | --- | --- | --- | --- | --- |
| 1.Your pain at its worst? |  |  |  |  |  |  |  |  |  |  |  |
| 2.Your fatigue (tiredness) at its WORST? |  |  |  |  |  |  |  |  |  |  |  |
| 3.Your nausea at its WORST? |  |  |  |  |  |  |  |  |  |  |  |
| 4. Your disturbed sleep at its WORST? |  |  |  |  |  |  |  |  |  |  |  |
| 5. Your feelings of being distressed (upset) at its WORST? |  |  |  |  |  |  |  |  |  |  |  |
| 6.Your shortness of breath at its WORST? |  |  |  |  |  |  |  |  |  |  |  |
| 7.Your problem with remembering things at its WORST? |  |  |  |  |  |  |  |  |  |  |  |
| 8.Your problem with lack of appetite at its WORST? |  |  |  |  |  |  |  |  |  |  |  |
| 9.Your feeling drowsy (sleepy) at its WORST? |  |  |  |  |  |  |  |  |  |  |  |
| 10.Your having a dry mouth at its WORST? |  |  |  |  |  |  |  |  |  |  |  |
| 11.Your feeling sadness at its WORST? |  |  |  |  |  |  |  |  |  |  |  |
| 12. Your vomiting sadness at its WORST? |  |  |  |  |  |  |  |  |  |  |  |
| 13.Your numbness or tingling at its WORST? |  |  |  |  |  |  |  |  |  |  |  |
| 14. Your weakness on one side of the body at its WORST? |  |  |  |  |  |  |  |  |  |  |  |
| 15. Your difficulty understading at its WORST? |  |  |  |  |  |  |  |  |  |  |  |
| 16. Your difficulty speaking (finding the words) at its WORST? |  |  |  |  |  |  |  |  |  |  |  |
| 17. Your seizures at its WORST? |  |  |  |  |  |  |  |  |  |  |  |
| 18. Your difficulty concentrating at its WORST? |  |  |  |  |  |  |  |  |  |  |  |
| 19. Your vision at its WORST? |  |  |  |  |  |  |  |  |  |  |  |
| 20. Your change in appearance at its WORST? |  |  |  |  |  |  |  |  |  |  |  |
| 21. Your change in boewl pattern at its WORST? |  |  |  |  |  |  |  |  |  |  |  |
| 22. Your irritability at its WORST? |  |  |  |  |  |  |  |  |  |  |  |

Part II How have your symptoms interfered with your life?

Symptoms frequently interfere with how we live. How much have your symptoms interfered with the following items in the last 24hrs? Please select a number from 0 (symptom has not been present) to 10 (the symptom was as bad as you can imagine it could be) for each item.

| Item | 0 | 1 | 2 | 3 | 4 | 5 | 6 | 7 |  | 8 | 9 | 10 |
| --- | --- | --- | --- | --- | --- | --- | --- | --- | --- | --- | --- | --- |
| 23.Genernal activity？ |  |  |  |  |  |  |  |  |  |  |  |  |
| 24.Mood？ |  |  |  |  |  |  |  |  |  |  |  |  |
| 25.Work (including work around the house)？ |  |  |  |  |  |  |  |  |  |  |  |  |
| 26.Relations with other people？ |  |  |  |  |  |  |  |  |  |  |  |  |
| 27.Walking？ |  |  |  |  |  |  |  |  |  |  |  |  |
| 28.Enjoyment of life？ |  |  |  |  |  |  |  |  |  |  |  |  |
